# Acceptability of pre-referral rectal artesunate for severe malaria in children under 5 years by health workers and caregivers in the Democratic Republic of the Congo, Nigeria and Uganda

**DOI:** 10.1186/s12936-022-04348-7

**Published:** 2022-11-10

**Authors:** Phyllis Awor, Joseph Kimera, Proscovia Athieno, Gloria Tumukunde, Jean Okitawutshu, Antoinette Tshefu, Elizabeth Omoluabi, Aita Signorell, Nina Brunner, Jean-Claude Kalenga, Babatunde Akano, Kazeem Ayodeji, Charles Okon, Ocheche Yusuf, Giulia Delvento, Tristan Lee, Christian Burri, Christian Lengeler, Manuel W. Hetzel

**Affiliations:** 1 School of Public Health, Makerere University College of Health Sciences, Kampala, Uganda; 2 Kinshasa School of Public Health, University of Kinshasa, Kinshasa, Democratic Republic of the Congo; 3 Akena Associates, Abuja, Nigeria; 4 Swiss Tropical and Public Health Institute, Allschwil, Switzerland; 5 University of Basel, Basel, Switzerland

## Abstract

**Background:**

For children below 6 years with suspected severe malaria attending a health care provider unable to provide parenteral malaria treatment, pre-referral rectal artesunate (RAS) is recommended by the World Health Organization to prevent death and disability. A number of African countries are in the process of rolling out quality-assured RAS for pre-referral treatment of severe malaria at community-level. The success of RAS depends, among other factors, on the acceptability of RAS in the communities where it is being rolled-out. Yet to date, there is limited literature on RAS acceptability. This study aimed to determine the acceptability of RAS by health care providers and child caregivers in communities where quality assured RAS was rolled out. This study was nested within the comprehensive multi-country observational research project Community Access to Rectal Artesunate for Malaria (CARAMAL), implemented in the Democratic Republic of the Congo (DRC), Nigeria, and Uganda between 2018 and 2020. Data from three different sources were analysed to understand RAS acceptability: interviews with health workers during three health care provider surveys (N = 341 community health workers and 467 primary health facility workers), with caregivers of children < 5 years of age during three household surveys (N = 9332 caregivers), and with caregivers of children < 5 years of age who were treated with RAS and enrolled in the CARAMAL Patient Surveillance System (N = 3645 caregivers).

**Results:**

RAS acceptability was high among all interviewed stakeholders in the three countries. After the roll-out of RAS, 97–100% heath care providers in DRC, 98–100% in Nigeria and 93–100% in Uganda considered RAS as very good or good. Majority of caregivers whose children had received RAS for pre-referral management of severe malaria indicated that they would want to get the medication again, if their child had the same illness (99.8% of caregivers in DRC, 100% in Nigeria and 99.9% in Uganda). In three household surveys, 67–80% of caregivers whose children had not previously received RAS considered the medication as useful.

**Conclusion:**

RAS was well accepted by health workers and child caregivers in DRC, Nigeria and Uganda. Acceptability is unlikely to be an obstacle to the large-scale roll-out of RAS in the studied settings.

## Background

Malaria is one of the leading causes of illness, death, and lost economic productivity globally. It still results in over 627,000 deaths each year, most of which are in children under 5 years of age in sub-Saharan Africa [1]. Most malaria deaths occur in remote settings where access to formal health facilities is poor and patients do not receive the required treatment and care in a timely manner [1]. Delays in obtaining treatment, particularly for severe disease, can quickly result in lasting sequelae or death. In situations in which parenteral treatment of severe malaria is not available, rectal artesunate (RAS) should be provided as pre-referral treatment for young children. Appropriately dosed, RAS rapidly (within 24 h) clears 90% or more of malaria parasites [2] and in a clinical trial it was shown to significantly reduce the risk of death or permanent disability in children less than 6 years of age, who cannot reach a health facility within six hours [3]. The World Health Organization (WHO), therefore, recommends treating children less than 6 years of age initially with a single rectal dose of 10 mg artesunate per kilogram of body weight, after which the child should be referred immediately to an appropriate health facility where the full treatment and care package can be provided [4].

In 2018, RAS became available for the first time from two WHO pre-qualified suppliers, and it is registered in many malaria-endemic countries, which are at some stage of using RAS as pre-referral treatment for severe malaria [4]. The successful large-scale roll-out of RAS depends, among other factors, on the product’s acceptability among caregivers and health care workers in the communities in which it is made available. A number of studies have found the rectal dosage form to be well accepted by parents of sick children in different cultural settings [5]. However, compliance and acceptability of rectal administration may depend on different factors specific to the local context [6, 7]. This study aimed to provide evidence of the acceptability of RAS in communities in the Democratic Republic of the Congo (DRC), Nigeria and Uganda in which RAS has been introduced on a large scale.

## Methods

### Study design

This study was conducted in the frame of the Community Access to Rectal Artesunate for Malaria (CARAMAL) project, a multi-country operational research accompanying the roll-out of quality assured RAS through established community-based health care providers in DRC, Nigeria and Uganda**.** In each of the countries, a continuous patient surveillance system (PSS) was set up and Health Care Provider Surveys (HCPS) and Household Surveys (HHS) were conducted annually from 2018 to 2020. More information on the project and research design can be found elsewhere [8].

### Study setting

The study was implemented in three Health Zones in DRC (Kenge, Kingandu and Ipamu); three Districts in Uganda (Apac, Kole and Oyam), and three Local Government Areas (Fufore, Mayo-Belwa and Song) in Adamawa State in Nigeria. The three project countries together share an estimated 42% of total global malaria cases and 39% of total malaria deaths. In the study areas, health care services are provided by community health workers (CHW) implementing integrated community case management (iCCM) programmes, primary health centres (PHC) and referral facilities with inpatient facilities [8].

### Study populations and data collection

#### Patient surveillance system

The PSS included children below 5 years of age seeking care for a severe febrile illness episode at the level of community-based care providers (CHW and PHC). Children enrolled in the PSS were followed up at their home 28 days after the initial visit to a CHW or PHC.

#### Health care provider survey

The HCPS included a random sample of health workers from all provider levels, including CHW, PHC and referral facilities surveyed in 2018, 2019 and 2020. The selection of the sample was meant to provide a good representation of all types of health workers who treat children with severe febrile illness in the study areas. This analysis was limited to data from CHW and PHC, the health care levels at which RAS is supposed to be available.

#### Household survey

For the HHS a stratified 3-stage cluster sampling strategy was utilized. Independent samples were selected annually. The stratification was by Health Zone, District or Local Government Area (LGA) for each of the three study countries, respectively, and the stages of cluster sampling included the zone/LGA/parish level, village level and household level. Clusters were sampled from the health zone/district in each country, with the number proportional to the population size. One village was then sampled from each cluster and within each village, 30 households were randomly sampled from a list of households with children < 5 years of age established by the survey team and village representatives prior to commencing data collection. A sampling interval was used to systematically include surveyed households. In each selected household, the household head and caregivers of children below 5 years of age were eligible to participate in the face-to-face structured interview. The person to be interviewed had to be an adult and able to answer questions about the household characteristics and/or health care of the children below 5 years in that particular household (i.e. usually the primary caregiver). More information on the survey methods can be found in Lengeler et al. and Awor et al. [8, 9].

The surveys were conducted at baseline (that is prior to introduction of RAS), at midline (about 1 year after introduction of RAS) and at endline (about 2 years after the introduction of RAS). Data was collected by face to face interviews. The interviews were conducted by teams of field interviewers who had received extensive training prior to commencing the survey. Quantitative data collected by field research teams was directly captured on internet/Wi-Fi capable tablets using Open Data Kit (ODK) electronic data collection software.

In the PSS, data was collected through face-to-face interviews with caregivers of children that had been enrolled, treated with pre-referral RAS and followed up 28 days later. For the HHS household heads and caregivers of children < 5 years in the household were interviewed. At each health facility, the field research team conducted an information session with the officer-in-charge upon arrival and, following this, with the health facility staff. Health facility staff who treat children were eligible for interview.

### Analysis

Results from the three data sources are provided, in order to give a holistic view of RAS acceptability among different stakeholders–the health care providers (using the HCPS); caregivers of young children (using the HHS) as well as caregivers who had direct experience of their child being treated with RAS (using the PSS). Basic knowledge, attitudes and practices related to RAS, among caregivers and service providers, are presented in this paper. Descriptive analyses of categorical outcomes were performed. For comparisons between years and background characteristics, chi-square statistics were used. Variables related to RAS perception were categorized and interpreted as follows: very good and good = “positive”, neutral, bad and very bad = “neutral/negative”.

### Ethics

The CARAMAL study protocol was approved by the Research Ethics Review Committee of the World Health Organization (WHO ERC, No. ERC.0003008), the Ethics Committee of the University of Kinshasa School of Public Health (No. 012/2018), the Health Research Ethics Committee of the Adamawa State Ministry of Health (S/MoH/1131/I), the National Health Research Ethics Committee of Nigeria (NHREC/01/01/2007–05/05/2018), the Higher Degrees, Research and Ethics Committee of the Makerere University School of Public Health (No. 548), the Uganda National Council for Science and Technology (UNCST, No. SS 4534), and the Scientific and Ethical Review Committee of CHAI (No. 112, 21 Nov 2017). Prior to any provider visit, the relevant local health authorities were informed. All interviews were conducted only after individual written informed consent.

## Results

### Characteristics of study participants

#### Household survey

Across all household surveys, 9332 caregivers of children < 5 years were interviewed. The average age of the caregivers in all the 3 countries was approximately 30 years and the majority of the caregivers were female. See Table 1 for more information on the household survey participants.

**Table 1:** Characteristics of care givers and health workers

Characteristics of the study populations									
	DRC			Nigeria			Uganda		
	PRE-RAS 2018	POST-RAS 2019	POST-RAS 2020	PRE-RAS 2018	POST-RAS 2019	POST-RAS 2020	PRE-RAS 2018	POST-RAS 2019	POST-RAS 2020
Household survey—caregiver characteristics									
	(N = 926)	(N = 947)	(N = 597)	(N = 1018)	(N = 1386)	(N = 1399)	(N = 1019)	(N = 1020)	(N = 1020)
Age (mean, SD)	34.5 (13.0)	30.5 (11.0)	30.5 (9.3)	30.1 (14.8)	29.9 (14.6)	30.1 (15.34)	30.9 (10.9)	31.2 (11.6)	30.9 (11.6)
Sex (female)	n/a	89.6	76.8	n/a	54.7	54.1	85.7	80.3	77.1
Health care provider survey—community health worker (chw) characteristics									
	n = 28	n = 42	n = 31	n = 40	n = 40	n = 40	n = 40	n = 40	n = 40
Age (mean, SD)	45.0 (10.6)	45.3 (10.4)	46.8 (9.2)	31.8 (7.0)	32.9 (8.5)	33.4 (7.7)	40.8 (10.7)	40.5 (8.9)	42.6 (9.7)
Sex (female)	10.7	4.8	9.7	22.5	52.5	52.5	32.5	25.0	47.9
Number of years as health worker: mean (SD)	7.8 (5.1)	8.3 (6.7)	7.8 (5.6)	2.9 (1.1)	3.9 (1.7)	4.5 (1.4)	10.0 (6.2)	11.1 (5.0)	11.1 (5.5)
Health care provider survey—primary health centre (PHC) staff characteristics									
	n = 65	n = 71	n = 37	n = 37	n = 55	n = 49	n = 41	n = 59	n = 53
Age (mean, SD)	44.0 (10.5)	44.1 (10.9)	43.9 (9.4)	40.2 (6.7)	42.8 (10.2)	39.9 (10.5)	34.8 (8.0)	36.9 (8.2)	36.9 (8.2)
Sex (female)	21.4	21.1	26.9	n/a	32.1	39.0	61.8	53.1	53.1
Number of years as health worker: mean (SD)	14.1 (9.1)	12.6 (15.1)	15.0 (9.9)	11.8 (6.7	16.7 (10.7)	12.2 (9.4)	9.1 (6.0)	11.6 (7.6)	11.6 (7.6)

#### Health care provider survey

Over the 3 survey rounds, 467 health workers at the PHC level were interviewed from all the countries. These health workers were about 40 years of age and they had worked for at least 10 years. About 40 CHWs were interviewed annually in each country, and these had been CHWs for at least 3 years (range 3–12 years) (Table 1).

#### Patient surveillance system

Of all the children enrolled in the patient surveillance system during the study period, 1721 received RAS as pre-referral treatment in DRC, 1780 in Uganda and 144 in Nigeria—Table 2. The interviewed caregivers of these children were mostly female at 73.6% and 84.8% for Nigeria and Uganda, respectively, while those of DRC were mostly male (61. 1%). They were mostly aged between 29 and 35 years. Most caregivers had attended some schooling, up to the primary or secondary school level, in all the three countries.

**Table 2:** Characteristics of caregivers whose child recieved RAS

Patient surveillance system			
Day 28 follow-up of severe malaria patients (2019-2020)			
	DRC	Nigeria	Uganda
Number of caregivers whose child received RAS	1721	144	1780
Age of caregiver (mean, SD)	31.2 (8.0)	34.7 (8.9)	29.3 (9.5)
Sex of caregiver (Female)	38.9	73.6	84.8
Highest level of school completed			
None	3.7	0.0	10.7
Preschool	3.9	0.0	41.3
Primary	23.0	31.2	37.2
Secondary	53.4	36.1	6.3
Some college/university	14.2	3.3	1.6
Vocational/technical school	1.8	n/a	2.9
Quranic	n/a	29.4	n/a

*n/a* not available (was not collected)

### RAS knowledge and acceptability by caregivers of children < 5 years

During the HHS it was found that knowledge of RAS was very minimal in the communities of the three countries at baseline and gradually improved during the study period. The percentage of caregivers who said they had heard of rectal artesunate, or artesunate suppository, for treating severe malaria, rose from 11.1 to 30.9% then to 62.1% in DRC, from 1.4 to 8.4% then to 15.8% in Nigeria and from 6.3 to 16.9% and eventually to 47.0% in Uganda (Table 3). The proportion of children that had received RAS in the communities was very low at baseline (before RAS was introduced) and only slightly increased towards the end of the study (to 4.8% in DRC, 1.2% in Nigeria and 5.3% in Uganda). A small proportion of caregivers had concerns about the use of RAS (Table 3). Some of the concerns related to the use of RAS that were raised included: possibility of side effects as the medicine was thought to be new, unavailability of the medicine and discomfort for the child, due to the rectal route of administration. Yet, overall, positive perceptions of RAS remained high across the study period, in all the 3 countries (67–78% in DRC, 78–80% in Nigeria and 72–88%) in Uganda.

**Table 3:** Knowledge and acceptability of RAS over 3 household survey rounds: % (95%CI)

	DRC			Nigeria			Uganda		
	PRE-RAS 2018	POST RAS 2019	POST RAS 2020	PRE-RAS 2018	POST RAS 2019	POST RAS 2020	PRE-RAS 2018	POST RAS 2019	POST RAS 2020
	(N = 926)	(N = 947)	(N = 597)	(N = 1018)	(N = 1386)	(N = 1399)	(N = 1019)	(N = 1020)	(N = 1020)
Caregiver has heard of RAS before									
Yes	11.1 (9.2,13.3)	30.9 (27.9,33.9)	62.1 (58.1,66.1)	1.4 (0.7,2.6)	8.4 (6.3,11.0)	15.8 (13.2,18.8)	6.3 (4.6,8.5)	16.9 (13.1,21.6)	47.0 (42.3,51.7)
Caregiver’s child (< 5 years) has received RAS befor**e**									
Yes	0.2 (0.0,0.8)	1.8 (1.0,2.9)	4.8 (3.2,6.9)	0.0 (0.0,0.4)	0.8 (0.4,1.4)	1.2 (0.7,2.0)	0.9 (0.5,1.7)	3.1 (2.2,4.3)	5.3 (3.2,8.6)
Caregiver has concerns giving rectal artesunate to their child									
Yes	13.7 (11.6,16.1)	11.7 (9.7,13.9)	5.4 (3.7,7.5)	n/a	2.5 (1.7,3.6)	2.4 (1.6,3.6)	3.5 (2.3,5.3)	1.3 (0.7,2.6)	1.1 (0.5,2.3)
Don't know	18.4 (15.9,21.0)	28.1 (25.3,31.1)	11.2 (8.8,14.1)	n/a	17.1 (14.8,19.7)	3.2 (2.3,4.5)	13.3 (10.6,16.4)	8.8 (6.6,11.7)	8.5 (6.7,10.7)
Caregiver thinks RAS is useful									
Yes	78.2 (75.4,80.8)	67.4 (64.3,70.4)	77.3 (73.8,80.7)	n/a	77.9 (75.1,80.4)	80.1 (77.4,82.5)	72.4 (67.3,76.9)	79.1 (75.1,82.6)	87.5 (84.8,89.8)
Don't know	17.8 (15.4,20.4)	28.1 (25.3,31.1)	16.8 (13.9,20.0)	n/a	20.7 (18.2,23.4)	15.2 (13.1,17.6)	23.4 (19.1,28.4)	11.5 (9.6,13.8)	9.5 (7.7,11.7)

*n/a* not available (was not collected)

### RAS knowledge and acceptability by health workers

In 2020, after RAS had been rolled out in the study areas, almost all health providers interviewed had heard about RAS—100% in DRC, 97.7% in Nigeria and 100% in Uganda among CHWs; and 100% in DRC, 97.8% in Nigeria and 98.1% in Uganda among health workers at PHCs. Most CHWs in DRC and Uganda (90.3% and 95.0% respectively) had dispensed RAS while only 44.3% of CHWs in Nigeria had done so by the time of the HCPS in 2020. In the same survey, most PHC facilities, that is 81.2% in DRC, 89.7% in Nigeria and 88.5% in Uganda had dispensed RAS. Most health workers interviewed had positive perceptions of RAS, considering it as either very good or good in 2020, this was 100% in DRC, 93% in Nigeria, and 90% in Uganda (Table 4).

**Table 4:** Health care provider knowledge and utilization of RAS

	CHW				PHC			
	PRE-RAS 2018	POST RAS 2019	POST RAS 2020	Chi Sq p value	PRE-RAS 2018	POST RAS 2019	POST RAS 2020	Chi Sq p value
DRC	**n = 28**	**n = 42**	**n = 31**		**n = 65**	**n = 71**	**n = 37**	
Heard about suppositories	64.3	100	96.8	< 0.001	53.1	95.5	100	< 0.001
Heard about RAS	100	97.6	100	0.492	95.2	100	100	0.046
RAS administered by CHW/used at facility	n/a	85.4	90.3	n/a	0.0	81.7	81.2	0.281
How do you feel about treating a sick child with a suppository?				< 0.001				< 0.001
Very good	14.3	42.9	90.3		17.1	55.6	69.2	
Good	60.7	57.1	6.5		60.0	41.1	30.8	
Neutral	25.0	0.0	3.2		18.6	3.3	0.0	
Bad	0.0	0.0	0.0		4.3	0.0	0.0	
Very bad	0.0	0.0	0.0		0.0	0.0	0.0	

NIGERIA	**n = 40**	**n = 40**	**n = 40**		**n = 37**	**n = 55**	**n = 49**	
Heard about suppositories	28.0	94.4	100.0	< 0.001	30.2	93.3	95.8	< 0.001
Heard about RAS	67.3	100.0	97.7	< 0.001	83.1	98.3	97.8	0.001
RAS administered by CHW/used at facility	n/a	20.4	44.3	n/a	0.0	89.5	89.7	0.288
How do you feel about treating a sick child with a suppository?				0.158				0.006
Very good	30.0	40.0	55.0		28.0	65.5	62.0	
Good	55.0	42.5	38.0		44.0	31.0	31.0	
Neutral	15.0	17.5	5.0		24.0	3.5	7.0	
Bad	0.0	0.0	0.0		4.0	0.0	0.0	
Very bad	0.0	0.0	2.0		0.0	0.0	0.0	

UGANDA	**n = 40**	**n = 40**	**n = 40**		**n = 41**	**n = 59**	**n = 53**	
Heard about suppositories	50.0	100	100	< 0.001	100	93.2	100	0.336
Heard about RAS	92.5	100	100	0.046	97.6	93.2	98.1	0.582
RAS administered by CHW/used at facility	n/a	85.0	95.0	n/a	67.5	87.3	88.5	0.872
How do you feel about treating a sick child with a suppository?				0.332				0.006
Very good	42.5	65.0	43		20.6	61.2	27	
Good	55.0	35.0	55		70.6	32.7	63	
Neutral	2.5	0.0	2		2.9	4.1	5	
Bad	0.0	0.0	0.0		5.9	2.0	5	
Very bad	0.0	0.0	0.0		0.0	0.0	0.0	

In DRC, the positive perception of RAS (as good or very good) increased among CHWs from 75% in the pre-RAS period to 97% post-RAS and among PHC providers from 77 to 100%. In Nigeria, the increasingly positive perception of RAS among CHWs and PHC was also observed. Whereas health workers in DRC were familiar with RAS even at the pre-RAS survey point (CHW: 100%, PHC: 95.2%), awareness of RAS increased significantly in Nigeria (CHW: from 67.3–97.7%, PHC: from 83.1–97.8%).

In Uganda, a majority of health workers had already heard about suppositories and RAS in particular, prior to the start of this project. After the roll out of RAS, almost all the health workers reported knowledge of RAS (See Table 4 for more information on RAS knowledge and acceptability by health workers).

### RAS acceptability by caregivers of children treated with pre-referral RAS

For the majority of sick children who had received RAS, their caregivers (96.6% in DRC, 97.9% in Nigeria and 85.9% in Uganda) reported this as their first time to receive the medication (Table 5).

**Table 5:** Acceptability of rectal artesunate by caregivers of children < 5 years with suspected severe malaria.

	DRC	Nigeria	Uganda
Total who received RAS (N)	1721	144	1780
Was it the first time the child received RAS?			
Yes	96.6	97.9	85.9
In your opinion, how well did this medicine work?			
Poor	12.7	2.8	1.5
Fair	1.7	1.4	1.4
Good	13.6	11.1	5.6
Very good	38.4	34.7	34.5
Excellent	33.7	50.0	57.0
In your opinion, what does this medicine do?			
Reduce severity of malaria	84.9	19.6	84.0
Reduce the risk of death	45.8	8.4	62.8
Reduce the risk of brain damage	11.1	100	20.3
Don't know	2.2	1.4	1.0
Would want to get RAS again if child had the same illness	99.8	100	99.9

Source—patient surveillance system

Almost all caregivers (99.8% in DRC, 100% in Nigeria and 99.9% in Uganda) of the sick children would desire their child to get RAS again if the child had the same illness. When asked about their opinion of how well RAS worked, in DRC, 33.7% of caregivers rated it as excellent, 38.4% as very good and 13.6% as good. In Nigeria, 50.0% rated RAS as excellent, 34.7% as very good and 11.1% as good. Similarly, in Uganda 57.1% rated the medicine as excellent, 34.5% rated it very good and 5.6% as good (Table 5).

The caregivers were also asked about their opinion regarding what the medicine does. Most of them reported that the medicine is used to reduce severity of malaria—that is 84.9% in DRC and 84.0% in Uganda. In Nigeria, the most frequently mentioned answer was that RAS is used to reduce the risk of brain damage (100%).

## Discussion

RAS acceptability was generally high among both CHWs and health workers at primary health care facilities. Two years after the introduction of RAS, 97–100% of health workers in DRC, 98–100% in Nigeria and 93–100% in Uganda considered RAS to be very good or good. These positive perceptions increased over time and with increasing experience of health workers in providing RAS, negative perceptions of RAS remained rare in all three study countries. The high acceptability may be attributed to the fact that there was already some use of non-quality assured RAS within the lower level health facilities, particularly in Uganda. Regardless, the study finding of very high proportions of health care providers accepting RAS and rating it as good or very good is encouraging.

RAS use in the communities, as assessed in the HHS, was initially very low and only minimally increased during the study period. This is expected as severe malaria cases are relatively rare events. Despite the low use of RAS, all caregivers interviewed (both those whose children had had an opportunity to receive RAS and those who had only heard about RAS) had very positive perception of RAS as a pre-referral treatment for severe malaria. The caregivers considered RAS to be very useful with many of them stating that RAS was being used to reduce severity of illness and prevent death among the children. Most of the caregivers whose children had previously received RAS were willing to have their child receive RAS if they had the same illness again. An increasing number of caregivers in the general population, the children of most of whom had never received RAS, also knew about RAS and had a generally positive perception about the usefulness of RAS.

A few recent studies have also shown high acceptability of RAS [5, 10]. These include one study on adherence to referral advice after use of RAS for treating children in DRC, where RAS acceptability was found to be 79%, 90% and 98% amongst mothers, CHWs and nurses, respectively [11]. Another study from Uganda in 2016 focused on high compliance with referral advice, after treatment of children with pre-referral RAS [12].

Acceptability of RAS as treatment for uncomplicated malaria was slightly lower, at 71%, in a study by Hinton et al. among caregivers in two sites in Papua New Guinea [6]. The main concerns related to perceived side effects of the rectal route or lack of spousal consent, especially among caregivers who had no prior knowledge of suppositories. Shame or hygiene were not significant concerns and caregivers whose children had received artesunate suppositories considered them highly effective and safe. A cross-sectional survey by Inthavilay et al. across Laos, where few families (< 10%) in the general population but 51% of health workers had experience treating children with suppositories, 96.8% of the surveyed families would agree to using rectal treatment for malaria but both health workers and family members considered the rectal administration less effective than other routes. Concerns amongst the caregivers related to the rectal route of administration included possible pain (28%), discomfort for the children (40%), difficult administration (34%) and possible side effects (20%) [13]. Our study did not find any strong concerns related to the rectal route of administration.

The strengths of the results presented in this paper are firstly the use of three complementary data sources, namely health care provider surveys, household surveys and the interviews with caregivers of children with suspected severe malaria who had been treated with RAS. The results from these data sources are consistent, showing high acceptability of RAS. Secondly, large data sets are utilized, with thousands of patients and caregivers included. Thirdly, data is presented from three countries with different cultural and health systems environments but a very high malaria burden. The consistent information obtained across the data sources would outweigh a possible recall bias.

## Conclusion

The results show high acceptability of using RAS in children with signs of severe malaria in DRC, Nigeria and Uganda, confirming limited evidence from previous studies. Acceptability is therefore unlikely to be an obstacle to the large-scale roll-out of RAS in a variety of cultural settings in sub-Saharan Africa.

## Data Availability

Data is available upon reasonable request from the authors and the CARAMAL consortium.
